# HDL-Mediated Cholesterol Efflux and Plasma Loading Capacities Are Altered in Subjects with Metabolically- but Not Genetically Driven Non-Alcoholic Fatty Liver Disease (NAFLD)

**DOI:** 10.3390/biomedicines8120625

**Published:** 2020-12-18

**Authors:** Alessia Di Costanzo, Annalisa Ronca, Laura D’Erasmo, Matteo Manfredini, Francesco Baratta, Daniele Pastori, Michele Di Martino, Fabrizio Ceci, Francesco Angelico, Maria Del Ben, Chiara Pavanello, Marta Turri, Laura Calabresi, Elda Favari, Marcello Arca

**Affiliations:** 1 Department of Translational and Precision Medicine, Sapienza University of Rome, 00161 Rome, Italy; laura.derasmo@uniroma1.it (L.D.); marcello.arca@uniroma1.it (M.A.); 2 Department of Food and Drug, University of Parma, 43124 Parma, Italy; annalisa.ronca@studenti.unipr.it (A.R.); elda.favari@unipr.it (E.F.); 3 Department of Chemistry, Life Science, and Environmental Sustainability, University of Parma, 43124 Parma, Italy; matteo.manfredini@unipr.it; 4 Department of Clinical, Internal, Anesthesiologic and Cardiovascular Sciences, Sapienza University of Rome, 00161 Rome, Italy; francesco.baratta@uniroma1.it (F.B.); daniele.pastori@uniroma1.it (D.P.); maria.delben@uniroma1.it (M.D.B.); 5 Department of Diagnostic of Radiological, Oncological and Anatomopathological Sciences, Sapienza University of Rome, 00161 Rome, Italy; micdimartino@hotmail.it; 6 Department of Experimental Medicine, Sapienza University of Rome, 00161 Rome, Italy; fabrizio.ceci@uniroma1.it; 7 Department of Public Health and Infectious Diseases, Sapienza University of Rome, 00161 Rome, Italy; francesco.angelico@uniroma1.it; 8 Centro Grossi Paoletti, Dipartimento di Scienze Farmacologiche e Biomolecolari, Università degli Studi di Milano, 20133 Milan, Italy; chiara.pavanello@unimi.it (C.P.); marta.turri@unimi.it (M.T.); laura.calabresi@unimi.it (L.C.)

**Keywords:** metabolic NAFLD, genetic NAFLD, reverse cholesterol transport (RCT), cholesterol efflux capacity (CEC), cholesterol loading capacity (CLC)

## Abstract

**Background**. Non-alcoholic fatty liver disease (NAFLD) increases the risk of atherosclerosis but this risk may differ between metabolically- vs. genetically-driven NAFLD. High-density lipoprotein (HDL)-mediated cholesterol efflux (CEC) and plasma loading capacity (CLC) are key factors in atherogenesis. **Aims**. To test whether CEC and CLC differ between metabolically- vs. genetically-determined NAFLD. **Methods**: CEC and CLC were measured in 19 patients with metabolic NAFLD and wild-type *PNPLA3* genotype (Group M), 10 patients with genetic NAFLD carrying M148M *PNPLA3* genotype (Group G), and 10 controls *PNPLA3* wild-types and without NAFLD. CEC and CLC were measured ex vivo by isotopic and fluorimetric techniques using cellular models. **Results**: Compared with Group G, Group M showed reduced total CEC (−18.6%; *p* < 0.001) as well as that mediated by cholesterol transporters (−25.3% ABCA1; −16.3% ABCG1; −14.8% aqueous diffusion; all *p* < 0.04). No difference in CEC was found between Group G and controls. The presence of metabolic syndrome further impaired ABCG1-mediated CEC in Group M. Group M had higher plasma-induced CLC than Group G and controls (*p* < 0.001). **Conclusions**: Metabolically-, but not genetically-, driven NAFLD associates with dysfunctional HDL-meditated CEC and abnormal CLC. These data suggest that the mechanisms of anti-atherogenic protection in metabolic NAFLD are impaired.

## 1. Introduction

Non-alcoholic fatty liver disease (NAFLD) is the most common liver disease affecting from 25% to 45% of general adult population [1]. It is characterized by the accumulation of neutral lipids (mainly triglycerides) within hepatocytes in the absence of significant alcohol consumption or other secondary causes [2]. Abdominal overweight/obesity, insulin resistance, type 2 diabetes mellitus (T2DM) and hypertriglyceridemia are the major determinants of NAFLD [3,4]. However, several genetic factors have also been implicated in the development of fatty liver [5], such as the I148M (rs738409) variant in the patatin-like phospholipase domain-containing protein 3 (*PNPLA3)* gene which showed the most clinically significant association [6].

Although NAFLD has the potential to cause liver-related morbidity and mortality, it is also strongly associated with an increased risk of atherosclerotic cardiovascular disease (ASCVD) [7,8,9]. In agreement, several proatherogenic mechanisms have been identified in patients with NAFLD [10]. However, it is unclear whether this association is related to excess fat liver storage *per se* or to the metabolic abnormalities that typically accompany NAFLD. By comparing subjects with NAFLD due to metabolic disturbances to those with genetic NAFLD due to *PNPLA3* GG genotype, we have found that the burden of carotid intima-media thickness (C-IMT) was higher in metabolically but not in genetically-driven NAFLD [11]. Accordingly, Castaldo et al. [12] reported that NAFLD associated with the presence of *PNPLA3* rs738409 variant was not consistently linked to C-IMT measures. Furthermore, by using a Mendelian randomization approach, Lauridsen et al. [13] demonstrated that the *PNPLA3* rs738409 was associated with an increased risk of chronic liver but not ischemic heart disease (IHD) [13]. The divergent association of metabolic and genetic NAFLD with ASCVD is not fully explained, although it could be linked differences in the functionality of anti-atherogenic mechanisms in these two forms of NAFLD.

It has been reported that high-density lipoprotein (HDL) may protect against atherosclerotic vascular damage [14]. It is thought that this action is mainly related to the ability of this lipoprotein in promoting the cholesterol efflux from tissues, the first step of the reverse cholesterol transport (RCT) process [15,16,17]. Cholesterol efflux involves members of the ATP-binding cassette (ABC) family of transporters, ABCA1 and ABCG1, interacting with different sub-fractions of HDL; ABCA1 exports unesterified cholesterol from cells to lipid free/poor ApoAI (mainly pre-β HDL particles), whereas ABCG1 delivers cholesterol to mature HDL particles (mainly α-HDL particles) [18]. Mature HDL are also involved in aqueous passive diffusion (PD) and scavenger receptor BI (SR-BI)-mediated efflux, a bidirectional facilitated transport of cholesterol that depends on the cholesterol concentration gradient [18]. Together, these pathways are responsible for the bulk of removal of cholesterol from macrophages, a cell type directly involved in atherogenesis [18]. On the other hand, also the macrophage cholesterol loading capacity (CLC), i.e., the overall ability of serum to load macrophages with cholesterol, has been reported to influence atherogenesis [19,20]. To date, few data are available on HDL functionality in subjects with NAFLD and none on CLC. In addition, the ability of HDL in performing CEC and CLC has never been explored in metabolically- vs. genetically-driven NAFLD.

Therefore, in this study we aimed to evaluate these parameters in subjects with metabolic NAFLD as compared to those with fatty liver primarily associated with genetic causes. Our hypothesis is that subjects with metabolic NAFLD may have dysfunctional HDL and augmented CLC, thus explaining their increased cardiovascular risk.

## 2. Material and Methods

### 2.1. Study Subjects

Twenty-nine patients with NAFLD and 10 control subjects without NAFLD were included in the present study. As previously reported [11], NAFLD patients were further divided into two groups according to the presence or absence of *PNPLA3* I148M polymorphism or known metabolic disturbances typically associated with NAFLD: (1) patients’ carriers of *PNPLA3* (I148I) wild-type genotype and metabolic alterations (N = 19, Group M; metabolic NAFLD); and (2) patient carriers of *PNPLA3* (M148M) homozygous genotype and without metabolic disturbances (N = 10; Group G, genetic NAFLD). Patients with NAFLD and controls were age and gender matched.

The clinical and biochemical characterization of enrolled subjects has been detailed elsewhere [11]. In brief, the presence of liver steatosis was confirmed by nuclear magnetic resonance with spectroscopy (MRS/MRI). Excess alcohol consumption was evaluated by the AUDIT questionnaire and the secondary cause of NAFLD was excluded according to standard clinical and biochemical criteria. Obesity was defined as a BMI > 30 kg/m^2^. Homeostasis model assessment (HOMA) was used to quantify insulin resistance (HOMA-IR). T2DM was diagnosed in accordance with WHO criteria [11]. The criteria recommended by the NCEP-ATP III Expert Panel of the US National Cholesterol Panel [21] were used to diagnose metabolic syndrome (MetS). At the time of enrolment, none of the patients was taking lipid lowering therapies [11].

Plasma total cholesterol, triglycerides, HDL cholesterol, apolipoprotein (apo) A-I and apoB were measured in the morning after overnight fasting, as previously reported [11,22]. Low-density lipoprotein (LDL)-cholesterol levels were calculated according the Friedewald’s formula. Liver enzymes (alanine transaminase [ALT], aspartate transferase [AST], gamma glutamyl transferase [γGT]), and HCV and hepatitis B virus antibodies were determined using standard procedures. A Roche/Hitachi COBAS CE 6000 analyzer (Roche Diagnostics, Switzerland) was used to determine plasma glucose and plasma insulin levels [11]. Plasma adiponectin, as a marker of metabolic disturbances [23], was measured by using a commercially available enzyme-linked immunoassay kit (R&D systems, Minneapolis, MI, USA).

A fluorogenic 5′-nucleotidase assay was used to identify the *PNPLA3* rs738409 C to G non-synonymous sequence variant, as previously reported [11,24,25,26].

The study protocol was reviewed and approved by the Ethics Committee of the University of Rome (ref. no: 2277, 13 October 2011). Written informed consent was obtained from all participants in accordance with the principles of the Helsinki Declaration.

### 2.2. Measurement of HDL-Mediated Cholesterol Efflux Capacity (CEC)

HDL-mediated CEC was examined using widely standardized isotope techniques. This purpose was achieved through the use of specific cell models whereby the main pathways of efflux of cellular cholesterol to HDL were evaluated [15,16,17,27]. For the evaluation of cholesterol aqueous diffusion (AD) efflux, J774 macrophage cells of murine derivation (J774 A.1, from ATCC) were used in basal conditions, while the incubation of the cells in the presence of 0.3 mM of a cAMP analog (cpt-cAMP, Sigma-Aldrich, Milan, Italy) allowed the induction of ABCA1 expression and were used as a study model for total CEC as previously described [28,29]. The difference between total CEC and AD mediated CEC made possible to identify the net contribution of cholesterol efflux mediated by ABCA1 [29]. Fu5AH cells, derived from rat hepatoma, were used to observe the efflux of cholesterol mediated by SR-BI in the presence or absence of an SR-BI inhibitor (Block Lipid Transfer-1 10 μM, ChemBridge, San Diego, CA, USA) [30]. In addition, to identify the cholesterol efflux mediated by ABCG1, CHO cells were used. CHO cells have been transfected or not with human ABCG1 gene. The difference between CEC of transfected cells and the non-transfected cells allowed to evaluate the contribution of ABCG1 [20,27]. Cells were initially labeled for 24 h with [1,2-3H] cholesterol (PerkinElmer, Milan, Italy) in the presence of an ACAT inhibitor allowing the maintenance of cholesterol in an unesterified form. The cells were then subjected to an equilibration period in a culture medium with 0.2% BSA (BSA, Sigma-Aldrich). Finally, for four hours cells were incubated with 2% serum for ABCA1, SR-BI, total and AD mediated CEC, while for the evaluation of ABCG1 mediated CEC the cells were incubated for 6 h at a concentration of 1% of serum [27]. CEC values were expressed as percentage ratio between the radioactivity released in the medium and the total radioactivity incorporated by the cells. In each experiment a pool of normo-lipidemic human sera was used as the reference standard. CEC values of these standard sera were used to normalize the different experiments so that inter-assay variability could be corrected [31]. Another pool of normo-lipidemic human sera were used in each experiment as the reference standard 2 and its CEC value, following normalization, was considered an index of intra-assay variability.

### 2.3. Measurement of Plasma-Mediated Macrophage Cholesterol Loading Capacity (CLC)

Human macrophages deriving from THP-1 have been used as a cell model for the evaluation of plasma CLC, as previously described [19]. THP-1 cells were seeded with the addition of 50 ng/mL Phorbol 12-Myristate 13-Acetate (PMA) (Sigma Aldrich, Milan, Italy) for 72 h to allow their differentiation into macrophages. For 24 h THP-1 cells were then incubated with 5% (*v*/*v*) of the patients’ whole serum. CLC is expressed as the ratio of the micrograms of cholesterol to the milligrams of protein. A fluorimetric test allowed the quantification of cellular cholesterol content (Amplex Red Cholesterol Assay Kit, Molecular Probes by Life Technologies, Eugene, OR, USA) in the cells subjected to lysis. The bicinchoninic acid method was used to measure protein concentration in cell lysates.

### 2.4. Analysis of HDL Subpopulations and Measurement of Plasma Cholesterol Esterification

The amount of circulating preβ-HDL has been reported to be an important determinant of HDL-dependent RCT [29]. Therefore, serum content of preβ-HDL was assessed by 2D electrophoresis followed by immunodetection against human ApoAI and expressed as a percentage of total ApoAI [32]. As plasma cholesterol esterification activity may also be related to HDL structure and function [32], we have calculated the ratio of plasma unesterified to total cholesterol (UC/TC) as an indirect measure of lecithin cholesterol acyl transferase (LCAT) activity in study subjects. To this aim, the plasma concentration of unesterified cholesterol was determined by enzymatic techniques, as reported [32].

### 2.5. Statistical Analysis

The study size was based on power calculations with at least 90% power to detect significant differences of 10% in CEC and 20% in CLC between group M and group G by Using G power software, version 3.1.9 [11].

The SPSS package (version 25.0) (SPSS, Inc., Chicago, IL, USA) was used for all statistical analyses. Data are reported as means and standard deviations (for normally distributed variables) or as medians (25–75th percentiles) (for non-normally distributed variables). Differences between groups were assessed with ANOVA or t-Student test for parametric variables or with Kruskal–Wallis and Mann–Whitney tests for non-parametric variables). The χ^2^ test was used to compare proportions. Correlations were evaluated by using Spearman correlation analysis. Non-normally distributed variables were transformed into natural logarithms before statistical analysis. Adjustments were made using the General Linear Model (GLM) and linear regression analysis when appropriate. *p* ≤ 0.05 were considered as statistically significant.

## 3. Results

### 3.1. Anthropometric and Metabolic Profiles of Study Groups

Baseline clinical and biochemical characteristics of study groups are summarized in Table 1. By selection, the three groups did not differ by age and gender distribution, but were significant different for BMI, waist circumference (WC), systolic and diastolic BP, HDL-C, total triglycerides, ApoAI, fasting glucose, and insulin levels, HOMA-IR and serum liver enzymes (all *p* ≤ 0.03). In Group M, T2DM and MetS were defined 15.8% and 42.1%, respectively; 15.8% of patients was taking hypoglycemic medications (metformin and repaglinide) and 61.1% antihypertensive drugs. When compared with patients in Group G and controls, those in Group M showed significantly lower levels of plasma adiponectin (all *p* < 0.001), further confirming the presence of metabolic disturbances in this group. Notably, Group G showed significantly reduced plasma HDL-C *(p =* 0.031) and ApoAI (*p* = 0.009) when compared with controls. As expected, the amount of hepatic fat content (HFF%) was markedly increased in NAFLD patients, but it was comparable between Group M and Group G. Also, after stratifying patients in Group M according to the presence of metabolic syndrome, we found that those with NAFLD and MetS were older (*p* = 0.026), showed higher BMI (*p* = 0.008) and WC (*p* = 0.021), higher fasting glucose (*p* = 0.012), and insulin levels (*p* = 0.012) as well as indices of insulin resistance (*p* = 0.008) when compared with those in Group M without MetS diagnosis (data not shown).

### 3.2. Comparison between Groups of HDL-Mediated Cholesterol Efflux Capacity (CEC)

**Figure 1:**
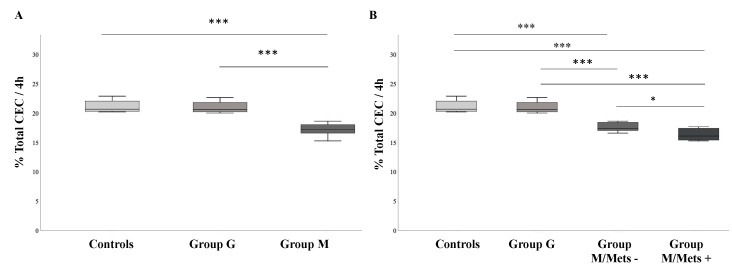
Whole HDL cholesterol efflux capacity according to NAFLD classification and presence of MetS. (**A**) shows differences in whole CEC according to NAFLD classification. In univariate analysis Group M had lower cholesterol efflux capacity independently from ApoAI levels and HFF% (*P*_adj_ < 0.001); (**B**) shows differences in whole CEC according to the presence of MetS. In univariate analysis Group M/MetS+ had lower CEC independently from age, ApoAI levels and HFF% (*P*_adj_ = 0.010) as compared to Group M/MetS−. Cholesterol efflux capacity expressed in relative efflux is presented in median and IQR. *** for *p* < 0.0001; * for *p* = 0.029. Group M, metabolic NAFLD group; Group G, genetic NAFLD group; Group M/MetS−, metabolic NAFLD group without MetS diagnosis; Group M/Mets+, metabolic NAFLD group with MetS diagnosis (see Section 2).

Total CEC from cAMP stimulated J774 macrophages was found significantly lower in Group M compared with Group G and controls (17.1 ± 1.1%, vs. 21.0 ± 0.9% vs. 21.1 ± 1.0%, respectively; all *p* < 0.001) (Figure 1, Panel A). As a result, patients with metabolic NAFLD showed an 18.6% reduction in total CEC when compared to those with genetic NAFLD and 19% when compared with controls (*p* < 0.001). Conversely, no difference in total CEC was found between Group G and controls. When patients in Group M were further categorized according to the presence of MetS, the relationship between metabolic NAFLD and reduced total HDL-mediated CEC was further confirmed. As shown in Figure 1, Panel B, a progressive decrease of total CEC was observed from patients in Group G to those in group M without MetS (Group M/MetS−) and to those with MetS (*p for trend* < 0.001).

**Figure 2:**
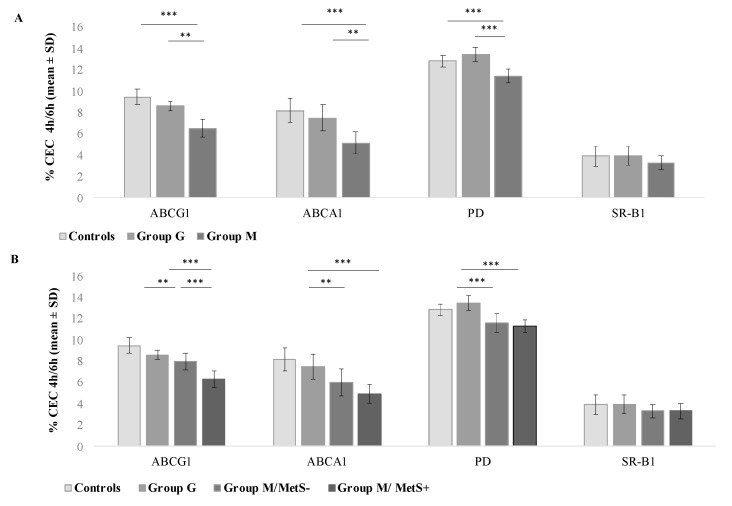
ABCG1-, ABCA1-, PD-, and SR-BI- mediated CEC according to NAFLD classification and MetS diagnosis. (**A**) shows differences in ABCA1-, ABCG1-, SR-BI- and PD-mediated CEC according to NAFLD classification; (**B**) shows differences in ABCA1-, ABCG1-, SR-BI-, and PD-mediated CEC according to the presence of MetS. Cholesterol efflux capacities expressed in relative efflux are presented in mean ± SD. *** for *p* < 0.0001; ** for *p* ≤ 0.002. ABCA1 indicates ATP binding cassette A1; ABCG1, ATP binding cassette subfamily G member 1; Group M, metabolic NAFLD group; Group G, genetic NAFLD group; Group M/MetS−, metabolic NAFLD group without MetS diagnosis; Group M/Mets+, metabolic NAFLD group with MetS diagnosis (see Section 2); SR-BI, scavenger receptor class B member 1.

By evaluating CEC occurring through the specific pathways, we found that the ABCG1-, ABCA1- and passive diffusion (PD)-mediated efflux, but not that mediated by SR-BI, was remarkably reduced in patients of Group M compared with those of Group G and controls (all *p* < 0.001) (Figure 2, Panel A). After dividing patients of Group M in those with and without MetS, a significant steady decrease in the ABCG1-mediated CEC was found from controls to Group G and from Group M/MetS− to Group M/MetS+ patients (*p for trend* = 0.031, Figure 2, Panel B). No differences emerged in CEC mediated by ABCA1 and SR-BI transporters and via PD between these groups.

**Figure 3:**
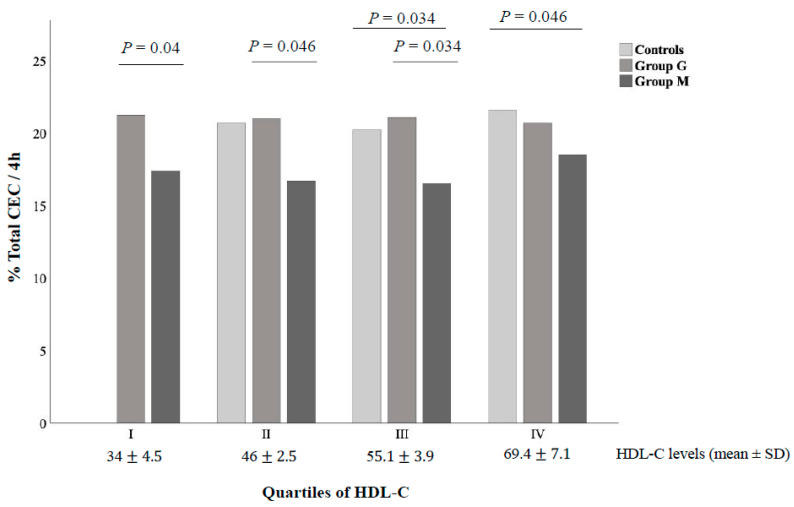
Whole CEC in study groups according to HDL-C levels. The figure shows differences in the whole cholesterol efflux capacity between groups according to quartiles of HDL-C. There were significant differences in efflux between all pairs with the same or similar HDL-C as determined by Mann-Whitney test. No Controls were present in the first HDL quartile.

As plasma HDL concentration was different between Group M and Group G (Table 1), we have compared CEC according to quartiles of plasma HDL-C levels (I quartile < 43 mg/dL, II quartile from 43 to 50.9 mg/dL, III quartile from 51 to 60.9 mg/dL and IV quartile ≥ 61 mg/dL). As reported in Figure 3, we found that plasma isolated from patients with metabolic NAFLD promoted significantly less cholesterol efflux from J774 compared to that obtained from patients with genetic NAFLD showing similar HDL-C levels (all *p* < 0.05). Conversely, no differences were found after comparing patients in Group G with controls.

**Figure 4:**
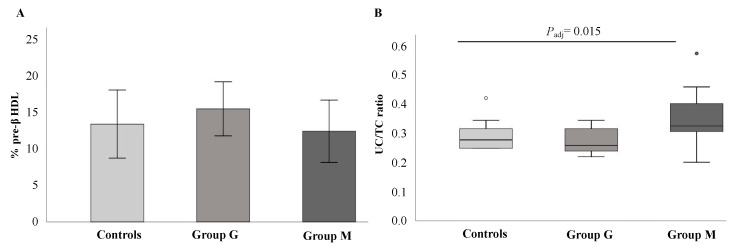
Comparison of percentage of pre-β HDL and UC/TC ratio between study groups. (**A**) shows pre-β HDL concentrations between study groups expressed as mean ± SE; (**B**) the box plot shows the ratio between unesterified cholesterol per total cholesterol (UC/TC ratio) expressed as median and IQR. *P*_adj_ = 0.015 adjusted for ApoAI levels and HFF%. Group M, metabolic NAFLD group; Group G, genetic NAFLD group.

To pursue more insight into the mechanism underlying the differences in CEC between metabolic and genetic NAFLD, we further compared HDL sub-fractions profile and plasma UC/TC ratio cholesterol between groups (Figure 4). As compared with patients with genetic NAFLD (Group G), those with metabolic NAFLD (Group M) showed a tendency towards lower pre-β HDL concentration (12.9 ± 5.9% vs. 16.2 ± 3.8%), even though the difference did not reach the statistical significance (Panel A). Conversely, we observed a significant higher UC/TC ratio in Group M when compared with the other groups. This difference persisted even after adjustment for ApoAI and HFF% (*P*_adj_ = 0.015) (Panel B).

### 3.3. Comparison between Groups of Plasma-Mediated Macrophage Cholesterol Loading Capacity (CLC)

**Figure 5:**
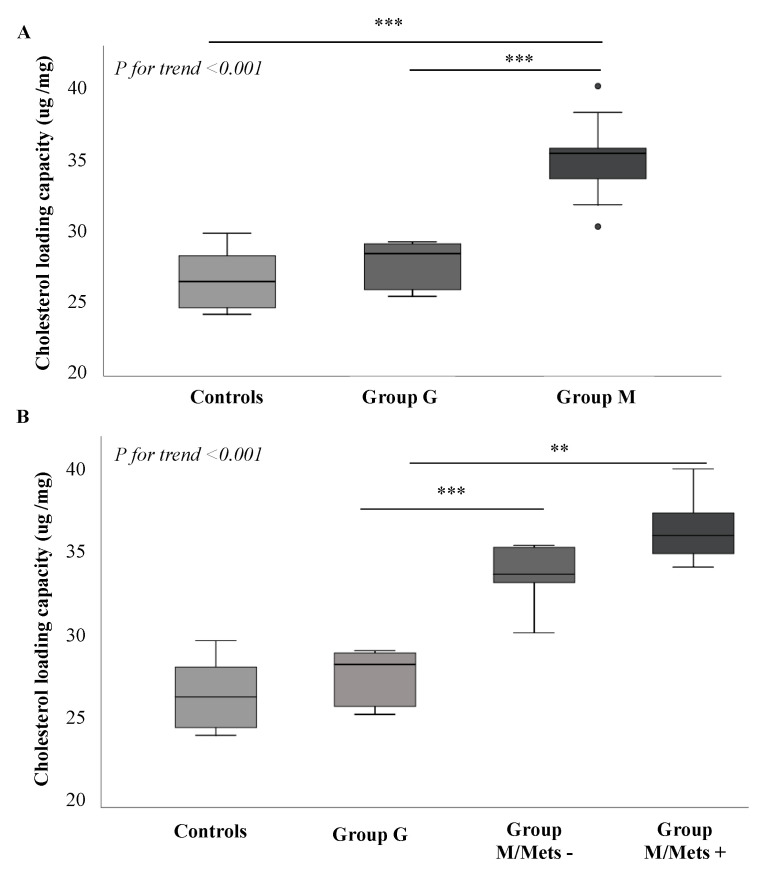
Comparison of plasma cholesterol loading capacity between study groups. (**A**) shows CLC according to NAFLD classification. *** *p* < 0.0001; (**B**) shows CLC according to MetS status. *** *p* < 0.0001; ** *p* = 0.003. Each bar represents the median and IQR cholesterol content of cells exposed to patient plasma. Group M, metabolic NAFLD group; Group G, genetic NAFLD group; Group M/MetS−, metabolic NAFLD group without MetS diagnosis; Group M/Mets+, metabolic NAFLD group with MetS diagnosis (see Section 2).

Cell cholesterol content is the result of both cholesterol efflux and influx [19]. Therefore, we decide to investigate the capacity of plasma isolated from study patients to load cholesterol into macrophages (CLC). As shown in Figure 5, Panel A, patients with metabolic NAFLD had the highest plasma induced CLC when compared with those with genetic NAFLD and controls (*p* < 0.001). After dividing Group M according to MetS, Group G still showed lower plasma-induced CLC (27.5 ± 1.5 μg/mg) when compared with Group M/MetS− (33.5 ± 1.6 μg/mg, *p* < 0.001), and Group M/MetS+ (35.9 ± 1.8 μg/mg, *p =* 0.003, Panel B).

### 3.4. Relationship of CEC and CLC with Clinical Parameters

**Figure 6:**
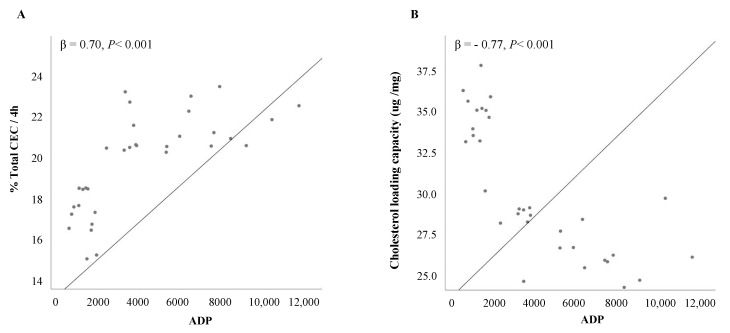
Linear regression analysis between CEC, CLC and adiponectin levels. (**A**) shows linear regression analysis between CEC and concentrations of adiponectin. R-squared = 0.49 (**B**) shows linear regression analysis between CLC and concentrations of adiponectin. R-squared = 0.60. ADP, adiponectin.

The correlations between total CEC and CLC with anthropometric and biochemical parameters in the whole population are given in Table S1. Notably, CEC negatively correlated with indices of obesity (r =- 0.61, *p* < 0.001) and HOMA-IR (r = −0.72, *p* < 0.001), systolic and diastolic BP (r = −0.41, *p* = 0.011 and r = −0.34, *p* = 0.042, respectively), total triglycerides (r = −0.52, *p* = 0.001), ALT levels (r = −0.57, *p* < 0.001) and HFF% (r = −0.44, *p* = 0.005). Conversely, CLC positively correlated with BMI (r = 0.43, *p* = 0.007) and HOMA-IR (r = 0.56, *p* < 0.001), systolic and diastolic BP (r = 0.59, *p* < 0.001 and r = 0.47, *p* = 0.004, respectively), total triglycerides (r = 0.59, *p* < 0.001), ALT levels (r = 0.55, *p* < 0.001) and HFF% (r = 0.52, *p* = 0.001). Interestingly, we found a strong linear association between concentrations of adiponectin with both total CEC (β = 0.70, *p* < 0.001) and CLC (β = −0.77, *p* < 0.001). Such relationships remained significant following adjustments for age, gender, smoking, BMI, HDL-C and HOMA-IR (all *P*_adj_ < 0.003) (Figure 6). Noteworthy, a significant inverse correlation was also noted between CEC and CLC in the whole cohort of study patients (β = −0.83, *p* < 0.001).

## 4. Discussion

The major finding of present study was the demonstration that patients with NAFLD due to metabolic features exhibit abnormalities in HDL-dependent CEC as well as in plasma CLC compared to patients with genetically- driven NAFLD and controls. Metabolic NAFLD patients with MetS diagnosis showed an even more evident deficiency in CEC and CLC. Overall, the increased ability to load cholesterol into human macrophages paralleled with HDL inability to remove it provides clear support to the existence of a proatherogenic unbalance in cellular cholesterol homeostasis in patients with metabolic NAFLD.

Two previous studies have investigated the relationship between HDL-mediated cholesterol efflux and hepatic steatosis [33,34]. In particular, Fadaei et al. [33] reported in patients with ultrasound defined NAFLD and cardio-metabolic risk factors a significant reduction of total CEC as compared with controls, with the ABCA1-mediated accounting for the large part of this reduction. However, the difference in the whole CEC disappeared after adjustments for the traditional metabolic risk factors, while ABCA1-mediated CEC persisted even after adjustments for confounders. Additionally, van den Berg et al. [34], found that the whole CEC was impaired in patients with NAFLD indirectly diagnosed based upon an increased fatty liver index (FLI ≥ 60). Interestingly this observation was independent from HDL levels and enhanced low-grade chronic inflammation.

Our findings extend these observations by considering additional pathways known to mediate cholesterol efflux from macrophages such as those involving the ABCG1 transporter, SR-BI and aqueous passive diffusion. Moreover, we have been able to estimate patients’ liver fat content very accurately by employing MRI/MRS. The observation that CEC was not impaired in patients where fatty liver was due to *PNPLA3* variant, which exhibited a hepatic fat content that was almost superimposable to that observed in patients with metabolic NAFLD, it is strongly supportive of the conclusion that the excess of hepatic fat, per se, did not impair HDLs functionality.

The explanation why HDL isolated from patients with metabolic NAFLD are less efficient in promoting cholesterol efflux may be related to the composition of these particles. We have been able to exclude any involvement of HDL cholesterol content, as the reduced ability of HDL from patients with metabolic NAFLD in promoting CEC was present within all quartiles of HDL-C. An alternative explanation may rely on the presence of some alteration in the qualitative composition of HDL population. The identification of a deficit in the ABCA1-mediated efflux, which is highly dependent from pre-β HDL particles [29] may be in favor of this hypothesis. Consistently, we found that patients with metabolic NAFLD tend to have lower concentration of this HDL subclass as compared to patients with genetic NAFLD. It is well known that unesterified cholesterol accepted by pre-β HDL is subsequently esterified by LCAT to form cholesteryl esters, which migrate into the core of HDL particles, resulting in the formation of mature α-HDL [18,29]. Indeed, we found significant higher levels of unesterified cholesterol in metabolic NAFLD patients suggestive of a deceleration of LCAT activity. This finding is in agreement with that reported by Fadei et al. [33] describing a positive correlation between CEC and LCAT activity. In addition, it might also explain why we have found in metabolic NAFLD a reduction of CEC mediated by ABCG1 and passive diffusion, which recognize mature HDL as the best cellular cholesterol acceptor [18]. Finally, the lack of SR-BI-mediated CEC in metabolic NAFLD is not surprising as this pathway is strongly dependent from the cholesterol concentration gradient between cell membrane and circulating acceptor [19]. Altogether, our data may suggest that the lower cholesterol efflux in metabolic NAFLD could be attributed to the reduction of pre-β HDL particles and to the lower LCAT activity, resulting into increased unesterified cholesterol and suppressed formation of mature α-HDL. Unfortunately, in our experiment we were not able to directly measure LCAT activity so that additional experiments are needed to definitively validate this hypothesis.

An additional interesting observation of our study was that MetS diagnosis was associated with a further impairment of the functionality of HDL. In fact, patients with NAFLD and MetS had the lowest whole efflux capacity, 7.4% lower than that observed in patients without MetS and 22% lower than that observed in *PNPLA3*-driven NAFLD group. Numerous studies have been conducted to evaluate the effect of MetS and insulin resistance on the ability of HDL to promote cholesterol efflux with controversial results. Some studies have reported higher CEC activity in patients with MetS and insulin resistance [35,36]. Conversely, other studies have highlighted that HDL isolated from patients having metabolic disorders display a panel of functional anomalies including reduced capacity to mediate efflux [37,38,39].

In a recent large cohort of 1202 patients with atherogenic dyslipidemia, Gall et al. [39] demonstrated that CEC was correlated with the presence of MetS and was reduced progressively by 4%–11% as a function of the increasing number of coexisting metabolic syndrome diagnostic criteria. Our findings are coherent with this previous observation and may be probably explained by differences in phospholipid HDL composition between patients with and without MetS, which has been reported to influence cholesterol efflux capacity [40]. In fact, by using lipidomic approach, Meikle et al. [41] observed negative associations between the two phospholipids contained in HDL particles (i.e., phosphatidylcholine and sphingomyelin) and the features of insulin resistance, such as DMT2 and/or a prediabetic status. Therefore, it is reasonable to hypothesize that changes in phospholipids due to MetS, might be involved in the further reduction of CEC in NAFLD patients with MetS. Furthermore, changes in phospholipids profiles, may also explain the strong reduction in these patients of ABCG1-pathway which is known to be dependent also from phospholipid plasma gradient [42]. Future research is needed to clarify these aspects.

Finally, as cellular cholesterol accumulation is the result of efflux-influx processes, we also measured the ability of patient’s’ plasma to load cholesterol into macrophages. We found a significant increase in serum macrophage CLC in metabolic NAFLD patients compared to those with *PNPLA3*-driven NAFLD. No previous study has investigated this mechanism in NAFLD. It is known that serum CLC depends mainly on LDL (which is the main cell cholesterol donor) and can be influenced by composition and physicochemical properties of these lipoprotein particles [19,43]. However, it must be noted the in our cohort CLC did not correlate with LDL-C concentration so that mechanisms underlying this finding remain uncertain.

Based on our data, it seems plausible that the impaired CEC and CLC we observed in metabolic but not in genetic NAFLD may constitute one possible link between metabolic fatty liver and atherogenesis as well as the reported divergent effect of the two NAFLD subtypes on cardiovascular risk. Due to the small sample size, we were not able to search for any association between HDL functionality and C-IMT. However, Fadei et al. [33] showed that impaired ABCA1-mediated CEC had an independent association with subclinical atherosclerosis in NAFLD patients. Furthermore, the strong correlation we found between CEC and CLC and plasma adiponectin, which exerts protective effects against vascular damage due to its anti-inflammatory and anti-atherogenic properties [44], may be well in line with this view.

Some limitations to this study should be acknowledged. We have investigated a small number of patients. However, a priori sample size analysis indicated that this number of samples for each group would allow detection of inter-group differences with a power of 95% in CEC and CLC measurements. It is also important to note that we did not evaluate the activity of other enzymes involved in HDL biology, i.e., CETP and PLTP, as well as LCAT. In addition, we did not consider actual cardiovascular events, thus limiting the predictive significance of these findings. Therefore, future research is needed to extent these results to large populations.

The results of present study may have some clinical implications. It gives further support to the notion of considering metabolic NAFLD as a separate entity within the NAFLD category. To this regard, it is worth to mention that a consensus of experts has recently proposed to use the definition of ‘metabolic dysfunction-associated fatty liver disease or MAFLD’ to identify the subtype of NAFLD where metabolic factors exert the predominant role [45]. In addition, our results also underline the importance of considering the disturbances in HDL-dependent cholesterol trafficking in cardiovascular projection strategies in patients with metabolic NAFLD.

## 5. Conclusions

Metabolically-, but not genetically-driven NAFLD associates with dysfunctional HDLs and abnormal cellular cholesterol trafficking. Disturbances of HDL-dependent cholesterol homeostasis in NAFLD may identify a subgroup of patients at higher risk of ASCVD.

## Figures and Tables

**Table 1:** Anthropometric and metabolic profiles of study groups.

		NAFLD		
	Controls	GroupG	GroupM	*p* Value
N	10	10	19	
Age, years	55.9 ± 6.9	54.0 ± 8.5	55.6 ± 3.8	0.7
Males, %	80	78.9	80	0.9
MetS, %	0	0 *	42.1	0.005
BMI, kg/m^2^	25.5 ± 2.9	27.2 ± 3.6	30.8 ± 5.8 ***	0.01
WC, cm	93.9 ± 9.4	99.0 ± 12.4	107.5 ± 10.1 ***	0.006
Smokers, %	50	40	15.8	0.12
T2DM, %	0	0	15.8	0.18
HFF, %	2.06 ± 1.61 **	36.1 ± 22.4	32.0 ± 19.6 ***	<0.001
Systolic BP, mmHg	115 (103.7–120)	120 (112.5–130) *	130 (120–140) ***	0.003
Diastolic BP, mmHg	70 (65–80)	80 (72.5–84.2)	80 (75–85) ***	0.015
Total cholesterol, mg/dL	206.8 ± 28.5	206.4 ± 35.2	208.2 ± 27.8	0.9
HDL cholesterol, mg/dL	64.2 ± 10.3 **	52.6 ± 11.8	45.6 ± 12.9 ***	0.002
LDL cholesterol, mg/dL	125.1 ± 30.2	133.1 ± 27.9	126.9 ± 24.5	0.7
Total triglycerides, mg/dL	85.5 (59.5–102.7)	88 (84.7–106) *	159 (125–210) ***	0.005
ApoB, mg/dL	97.9 ± 16.5	97.5 ± 16.5	92.0 ± 21.3	0.6
ApoAI, mg/dL	133 ±14.4 **	113.9± 14.6	106.6 ± 20.0 ***	0.002
Fasting glucose, mg/dL	78.5 ± 10.0	82.9 ± 13.8	98.0 ± 21.2 ***	0.01
Fasting insulin, U/L	5.3 (4.2–7.9)	5.9 (4.9–10.9) *	12.7 (8.6–18.9) ***	0.01
HOMA -IR	0.9 (0.9–1.6)	1.2 (0.9–2.2) *	2.9 (1.8–4.2) ***	<0.001
ADP, ng/dl	6596.8 ± 3179.6	4791.5 ± 1796.4 *	901.0 ± 413.8 ***	<0.001
AST, U/L	29.5 (19.7–36.5)	31.5 (22.2–33.2) *	20 (18–26)	0.026
ALT, U/L	13 (10–22)	16 (13.7–19.5) *	34 (21.5–42.5) ***	<0.001
y-GT, U/L	18.5 (13.2–24.5)	20.5 (15.2–29.2)	25.5 (20.7–38.2) ***	0.05

Data are shown as mean ± SD for normal distributed variables and median (inter quartile range) for non-normal distributed variables. * *p* < 0.05 for comparison between group M vs. group G; ** *p* < 0.05 for comparison between group G vs. controls; *** *p* < 0.05 for comparison between group M vs. controls. ADP, adiponectin; ApoAI, apolipoprotein A-I; BMI, body mass index; HDL-C, high-density lipoprotein cholesterol; AST, aspartate aminotransferase; ALT, alanine aminotransferase; HFF, hepatic fat fraction; HOMA-IR, homeostasis model assessment of insulin resistance; BP, blood pressure; MetS, Metabolic syndrome; T2DM, Type 2 diabetes; WC, waist circumference.

## Supplementary Materials

The following are available online at https://www.mdpi.com/2227-9059/8/12/625/s1. Table S1. Spearman correlation coefficients of HDL cholesterol loading capacity (CLC) and HDL cholesterol efflux capacity (CEC) with anthropometric and biochemical variables in the whole population.
