# Supplementary material for: HDL-Mediated Cholesterol Efflux and Plasma Loading Capacities Are Altered in Subjects with Metabolically- but Not Genetically Driven Non-Alcoholic Fatty Liver Disease (NAFLD)
Source: Biomedicines. 2020 Dec 18;8(12):625. doi: 10.3390/biomedicines8120625 (PMC7766839; doi:10.3390/biomedicines8120625)
Supplement: Supplementary file 1 [file biomedicines-08-00625-s001.pdf]

Table S1. Spearman correlation coefficients of HDL cholesterol loading capacity (CLC) and HDL cholesterol efflux capacity (CEC) with anthropometric and biochemical variables in the whole population.

	**Cholesterol loading capacity (CLC)** r (*P-value*)	**Total Cholesterol efflux capacity (CEC)** r (*P-value*)
**BMI**	0.43 (P=0.007)	-0.61 (P=0.001)
**WC**	0.46 (P=0.004)	-0.61 (P<0.001)
**Systolic BP**	0.59 (P<0.001)	-0.41 (P=0.011)
**Diastolic BP**	0.47 (P=0.004)	-0.34 (P=0.042)
**ApoA-I**	-0.45 (P=0.005)	0.38 (P=0.019)
**HDL**	-0.50 (P=0.001)	0.47 (P=0.003)
**Total triglycerides**	0.59 (P<0.001)	-0.52 (P=0.001)
**Fasting glucose**	0.49 (P=0.002)	-0.49 (P=0.002)

**Fasting Insulin**	0.53 (P=0.001)	-0.69 (P<0.001)
**HOMA-IR**	0.56 (P<0.001)	-0.72 (P<0.001)
**ALT**	0.55 (P<0.001)	-0.57 (P<0.001)
**AST**	-0.44 (P=0.005)	0.40 (P=0.011)
**HFF%**	0.52 (P=0.001)	-0.44 (P=0.005)
**ADP**	-0.81 (P<0.001)	0.78 (P<0.001)

r, correlation coefficient; ADP, adiponectin; apoA-I, apolipoprotein A-I; BMI, body mass index; HDL-C, high-density lipoprotein cholesterol; AST, aspartate aminotransferase; ALT, alanine aminotransferase; HFF, hepatic fat fraction; HOMA-IR, homeostasis model assessment of insulin resistance; BP, blood pressure; WC, waist circumference.

Spearman correlation analysis. Only significant associations were reported.
